# Top Skin-of-Color Publications in Dermatology

**DOI:** 10.2196/37256

**Published:** 2022-06-06

**Authors:** Benjamin R Cooper, Jaclyn B Anderson, Melissa R Laughter, Colby L Presley, J Mark Albrecht, Robert P Dellavalle

**Affiliations:** 1 College of Osteopathic Medicine Rocky Vista University Parker, CO United States; 2 Department of Pathology Stanford University Stanford, CA United States; 3 Dell Medical School The University of Texas at Austin Austin, TX United States; 4 Division of Dermatology Lehigh Valley Health Network Allentown, PA United States; 5 School of Medicine University of Utah Salt Lake City, UT United States; 6 Department of Dermatology University of Colorado Anschutz Medical Campus Aurora, CO United States; 7 Department of Epidemiology Colorado School of Public Health University of Colorado Anschutz Medical Campus Aurora, CO United States; 8 Dermatology Service Rocky Mountain Regional Veterans Affairs Medical Center Aurora, CO United States

**Keywords:** skin of color, dermatology, Web of Science, Altmetric, Altmetric Attention Score, decision-making, public attention, media, blogs, skin disorder, dermatologic conditions, online media, publication, citation, impact, health information, information exchange, education

## Introduction

Tools such as the Altmetric Attention Score (AAS) and Web of Science (WoS) allow researchers to qualify their work’s impact. WoS uses publication citation counts and is often utilized in academia, while the AAS analyzes online media attention to determine the impact of research [1].

## Methods

Using Altmetric Explorer to obtain an article’s AAS and WoS to measure an article’s citation count, the top 50 papers from each search engine were collected using the keywords “skin of color” and “dermatology.” An article’s mention in news outlets and on blogs and Twitter were recorded from Altmetric Explorer as well as whether these mentions came from members of the public or practitioners; this we defined as metrics indicative of online media “attention.” The mean (SD), 95% CI, and *P* values were determined by comparing the metrics provided by the top 50 papers from Altmetric and WoS.

## Results

Table 1 presents a comparison of the top 10 cited articles on WoS and the top 10 articles with the highest AAS.

The mean AAS for the top 50 papers from Altmetric and WoS were 39.1 and 24.2, respectively (*P*=.02). The *P* values were statistically significant in several of the categories compared, including the AAS (*P*=.02), news outlet mentions (*P*=.008), and Twitter mentions (*P*=.02) (Table 2). Recurring themes in the top AAS skin-of-color (SoC) papers included skin cancer; cosmetic dermatology, notably pigmentation disorders; and inadequate knowledge among health care practitioners in identifying dermatologic conditions in SoC patients. By contrast, the top-cited SoC papers from WoS involved basic science research of dermatologic conditions and recommendations for assessment tools for clinicians and patients.

**Table 1:** Top 10 cited dermatology papers on Web of Science compared to the top 10 high-scoring Altmetric papers.

Article title		Authors	Altmetric Attention Score	Total citations (on Web of Science), n	Publication year	Journal	Country
**Top 10 Altmetric articles**							
	Postinflammatory hyperpigmentation: A review of the epidemiology, clinical features, and treatment options in skin of color	Davis et al [2]	211	359	2010	JCAD^a^	United States
	Acne vulgaris in skin of color: Understanding nuances and optimizing treatment outcomes	Alexis [3]	179	12	2014	JDD^b^	United States
	Background and room illumination in color identification of skin lesions	Maymone et al [4]	128	6	2017	*JAMA* ^c^ *Dermatology*	United States
	Skin cancer and photoprotection in people of color: A review and recommendations for physicians and the public	Agbai et al [5]	122	120	2014	JAAD^d^	United States
	UV Exposure and the risk of cutaneous melanoma in skin of color: A systematic review	Lopes et al [6]	102	7	2021	*JAMA Dermatology*	United States
	Skin color in dermatology textbooks: An updated evaluation and analysis	Adelekun et al [7]	98	17	2021	JAAD	United States
	Skin cancer in skin of color	Bradford [8]	80	196	2009	*Dermatology Nursing*	United States
	The use of noncultured regenerative epithelial suspension for improving skin color and scars: A report of 8 cases and review of the literature	Ren et al [9]	72	2	2019	*Journal of Cosmetic* *Dermatology*	China
	Gaps in the understanding and treatment of skin cancer in people of color	Kailas et al [10]	54	4	2016	JAAD	United States
	How dermatology is failing melanoma patients with skin of color: Unanswered questions on risk and eye-opening disparities in outcomes are weighing heavily on melanoma patients with darker skin	Nelson [11]	53	3	2020	*Cancer Cytopathology*	United States
**Top 10 Web of Science articles**							
	Skin cancer in skin of color	Gloster Jr et al [12]	26	301	2006	JAAD	United States
	Skin of color: Biology, structure, function, and implications for dermatologic disease	Taylor [13]	34	220	2002	JAAD	United States
	A mouse model of vitiligo with focused epidermal depigmentation requires IFN-gamma for autoreactive CD8(+) T-cell accumulation in the skin	Harris et al [14]	24	159	2012	JID^e^	United States
	Skin cancer and photoprotection in people of color: A review and recommendations for physicians and the public	Agbai et al [5]	122	120	2014	JAAD	United States
	Acne vulgaris in skin of color	Taylor et al [15]	2	102	2002	JAAD	United States
	The Asian dermatologic patient review of common pigmentary disorders and cutaneous diseases	Ho et al [16]	1	84	2009	*American Journal of Clinical Dermatology*	China
	Melasma: an up-to-date comprehensive review	Ogbechie-Godec et al [17]	10	61	2017	*Dermatology and Therapy*	United States
	Development and validation of a vitiligo-specific quality-of-life instrument (VitiQoL)	Lilly et al [18]	2	61	2013	JAAD	United States
	Accuracy of self-report in assessing Fitzpatrick skin phototypes I through VI	Eilers et al [19]	18	53	2013	*JAMA Dermatology*	United States
	Defining pseudofolliculitis barbae in 2001: A review of the literature and current trends	Perry et al [20]	3	52	2002	JAAD	United States

**Table 2:** The top 50 Altmetric papers versus the top 50 cited papers in Web of Science.

	Altmetric Attention Score	Citations	News outlet mentions	Blog mentions	Twitter mentions	Count of mentions by members of the public	Count of mentions by practitioners
Top 50 skin-of-color Altmetric publications, mean (95% CI)	39.1 (27.1-51.1)	41.9 (20.2-63.6)	4.7 (3.0-6.4)	0.2 (0.1-0.4)	9.9 (6.2-13.6)	5.6 (3.5-7.8)	1.0 (0.5-1.5)
Top 50 skin-of-color Web of Science publications, mean (95% CI)	24.2 (14.6-33.8)	46.1 (31.2-61.0)	2.1 (1.1-3.2)	0.2 (0.1-0.4)	5.9 (2.9-8.8)	4.1 (2.0-6.2)	0.7 (0.2-1.2)
*P* value^a^	.02	.34	.008	.42	.02	.17	.11

^a^JCAD*: Journal of Clinical & Aesthetic Dermatology*.

^b^JDD: *Journal of Drugs in Dermatology*.

^c^JAMA: *Journal of the American Medical Association*.

^d^JAAD: *Journal of the American Academy of Dermatology*.

^e^JID: *Journal of Investigative Dermatology.*

^a^*t* test.

## Discussion

### Principal Findings

While highly cited publications often guide clinical recommendations and carry substantial influence on practitioners, they may fail to highlight the discussions taking place outside of the scientific community [21]. For SoC patients, their interests and concerns regarding dermatologic conditions must be understood by health care providers as disease processes often manifest differently in this population compared to the general public [13]. With over 70% of the US population using social media, these platforms will allow increased sharing of research topics, supporting the utility of Altmetric scoring compared to citation count alone [22]. Within our study, the difference in recurrent themes between top AAS versus top-cited publications indicated that the clinical mindset and patient-centered topics may not align.

### Limitations and Future Work

Limitations to our study include a small sample size, narrow inclusion criteria, and a lack of time constraints. Future studies comparing AAS and WoS should be confined to a short time period to mitigate temporal confounding factors due to the differing accrual rates of citation count and AAS [23]. Medical societies and health care providers can use insights from this study to shape the practice of dermatology to better understand the interests and expectations of SoC patients.

### Conclusion

AAS and WoS provide different metrics on the influence of academic research. Factors that may generate greater social media attention include papers with more pictures and an author’s social media presence. Elements that may produce greater citation counts include a journal’s impact factor and an author’s academic reputation and home institution. Altmetric uniquely represents the attention of the general public, which can facilitate patient-centered decision-making.

## Abbreviations

AAS: Altmetric Attention Score
SoC: skin of color
WoS: Web of Science
